# Eradication of advanced pelvic hydatid bone disease after limb salvage surgery – 5-year follow-up: a case report

**DOI:** 10.1186/1752-1947-9-21

**Published:** 2015-04-21

**Authors:** Muhammad Shahid Khan, Pervaiz Mehmood Hashmi, Dawar Khan

**Affiliations:** 1 Present Address: Department of Orthopaedics, Shah Bhitai District Government Hospital, Hyderabad, Pakistan; 2 Head Section of Orthopaedics, Department of Surgery, Aga Khan University Hospital, Karachi, Pakistan; 3 Department of Radiology, Aga Khan University Hospital, Karachi, Pakistan

**Keywords:** Hydatid bone disease, Limb salvage surgery

## Abstract

**Introduction:**

Echinococcosis is produced by the larval stage of *Echinococcus granulosus*; it is a parasitic disease which is seen rarely in humans and has adverse outcomes. We report a case of advanced pelvic hydatid bone disease with successful limb salvage surgery. Our patient had a 5-year follow-up without recurrence which is a rarity as per the literature. Early diagnosis and prompt medical therapy are necessary for effective management whereas delayed diagnosis is always fraught with the risk of recurrence and sepsis.

**Case presentation:**

In 2009, a 30-year-old woman, native of Karachi (Sindhi ethnicity), presented at our clinic with history of a pathological fracture 11 years earlier. Her fracture was initially misdiagnosed and fixed. Subsequently she had persistent disease that progressed with time. Following this she underwent multiple surgeries and the diagnosis of hydatid disease was made but despite multiple debridements and medical therapy she was not cured and finally she was offered a hemipelvectomy (limb sacrifice). On presentation to our hospital she was counseled regarding options of hemipelvectomy versus a limb salvage form of modified internal hemipelvectomy and wide margin resection. She opted for limb salvage. She underwent internal hemipelvectomy with wide margin resection of soft tissue and proximal femur along with postoperative albendazole therapy. She was able to walk again after a very long period. Currently she is 5-years postreconstructive surgery. She is infection free and ambulant without support.

**Conclusions:**

Hydatid bone disease is a rare entity in our part of the world but a careful history and thorough look at the initial images of our patient would have led to the suspicion of pathologic fracture and subsequent early diagnosis of this difficult problem. A second important learning point in this case was the lack of early referral to a center where this difficult problem could have been handled effectively. This could have minimized the physical, mental and financial stress to the patient and her family.

## Introduction

Hydatid cyst disease is a parasitic disease caused by a cestode known as echinococcus. The genus *Echinococcus* includes three species of which *Echinococcus granulosus* is the most common cause of hydatid disease in humans [1–3]. The tapeworm resides in the small bowel of the hosts and infected ova are shed in the feces. When ingested by intermediate hosts such as humans, sheep, or cattle, the larvae enter the portal circulation. The larvae eventually reach the liver; where most of them are trapped. Sometimes, larvae reach the lungs and other areas of the body and form cysts. The strong structure of osseous tissue limits the growth of the hydatid cyst, which spreads along medullar and trabecular channels [4]. The disease affects the long bones, vertebral column, pelvis, and costae in order from least to most affected region [1, 5]. Hydatid bone disease is rare, approximately 0.5 to 2.5% of all human hydatidosis [6]. Although long-term survival is possible, the disease is not easy to eradicate and may be impossible to cure [7–9]. Hydatid bone disease is essentially the disease of the young. Early diagnosis is primarily based on X-ray findings which are not specific to the disease. Patients usually present at an advanced stage of the disease and, therefore, treatment is difficult and recurrence is common. It is a serious disease which is difficult to eradicate because of complex and difficult resection. We present a patient who was initially misdiagnosed and underwent multiple surgeries. She is in our follow-up (more than 5 years now) and until now she is infection and disease free.

## Case presentation

**Figure 1:**
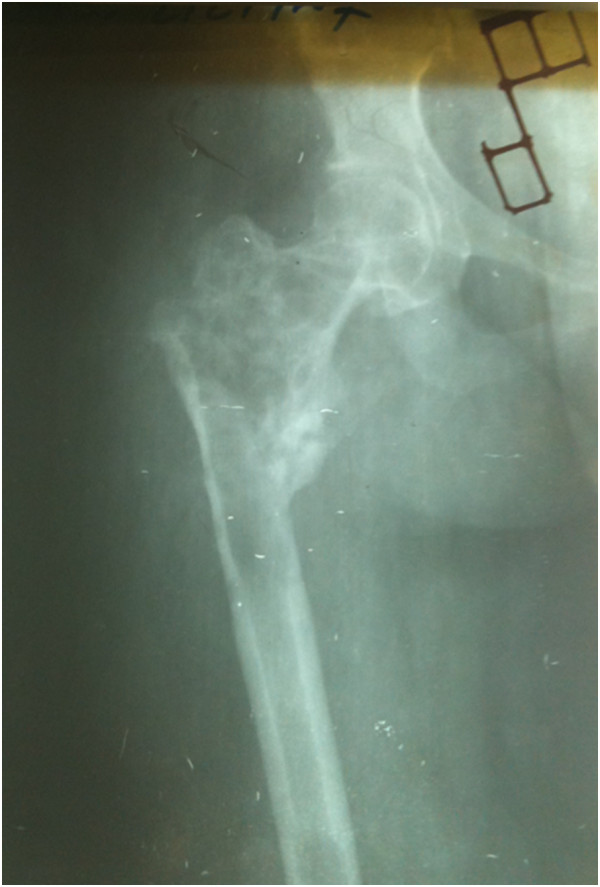
Initial X-ray of the patient before surgical intervention in 1998. There is proximal femur fracture along with radiolucencies present in proximal femur including the femoral head.

**Figure 2:**
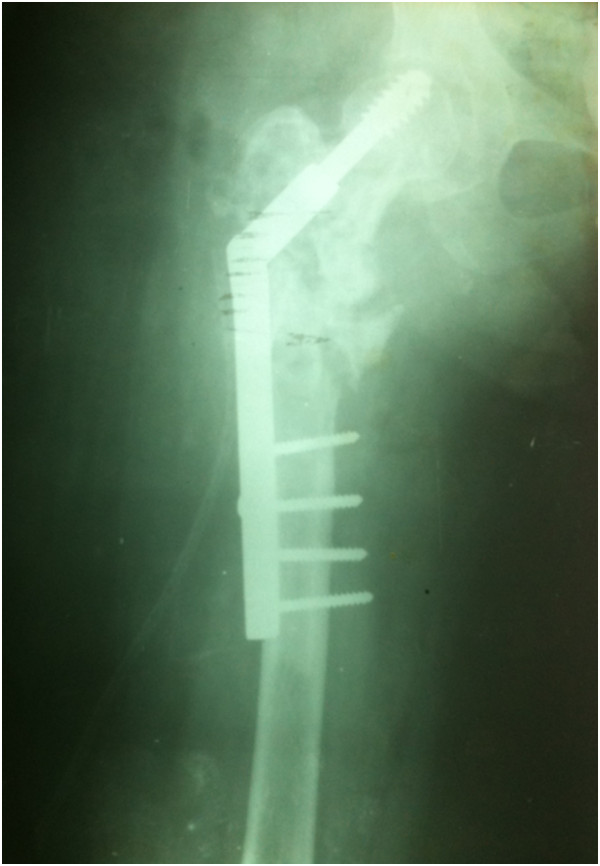
Initial fixation with dynamic hip screw done in 1998.

**Figure 3:**
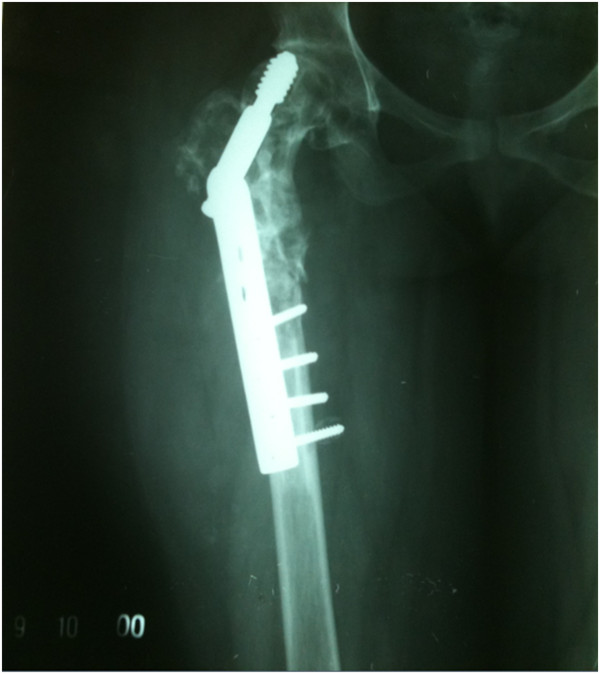
Further progression of radiolucencies in proximal femur along with lag screw cutting through the femoral head. Year 2000.

**Figure 4:**
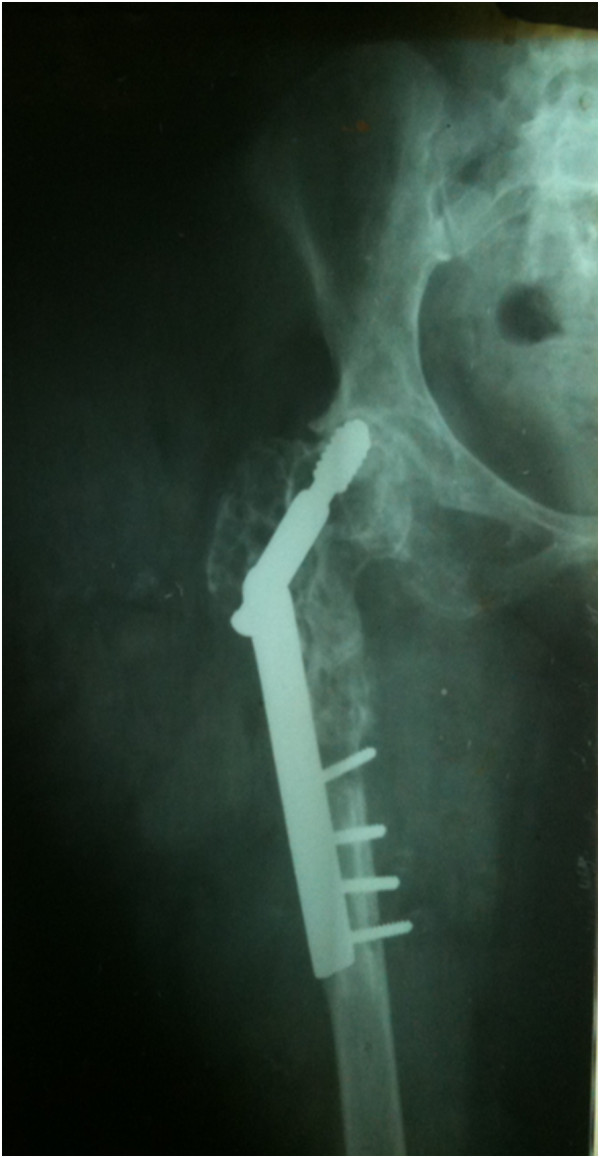
Further progression of disease involving acetabulum as well. Year 2007.

**Figure 5:**
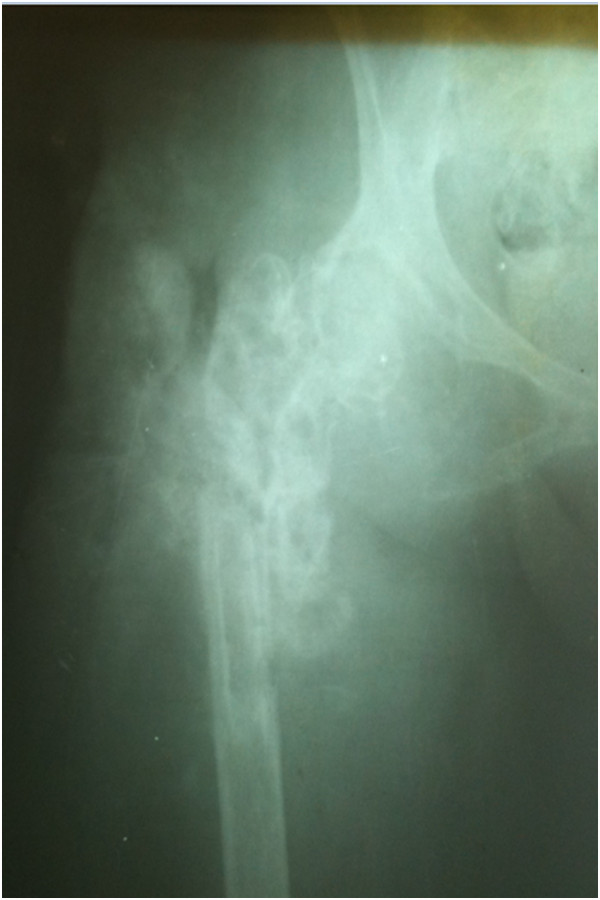
Implant removal done in year 2007.

In 2009, a 30-year-old woman, native of Karachi (Sindhi ethnicity), presented at our clinic with a 3-year history of discharging sinus of her right hip and inability to ambulate. She had a history of right proximal femur fracture secondary to a trivial trauma 11 years earlier (Figure 1). She initially remained under treatment of bone setters for 3 months and then she underwent fixation (in 1998) with dynamic hip screw (Figure 2) in a tertiary hospital without a biopsy being done although history and X-rays were suggestive of pathologic fracture. She remained symptomatic postoperatively and gradually the implant cut through the femoral head (Figures 3 and 4). In 2007 the implant was removed (Figure 5) in another tertiary hospital and tissue was sent for histopathology which showed hydatid disease. Postoperatively she developed discharging sinus for which she underwent multiple debridements along with prolonged courses of albendazole but her condition did not improve.

**Figure 6:**
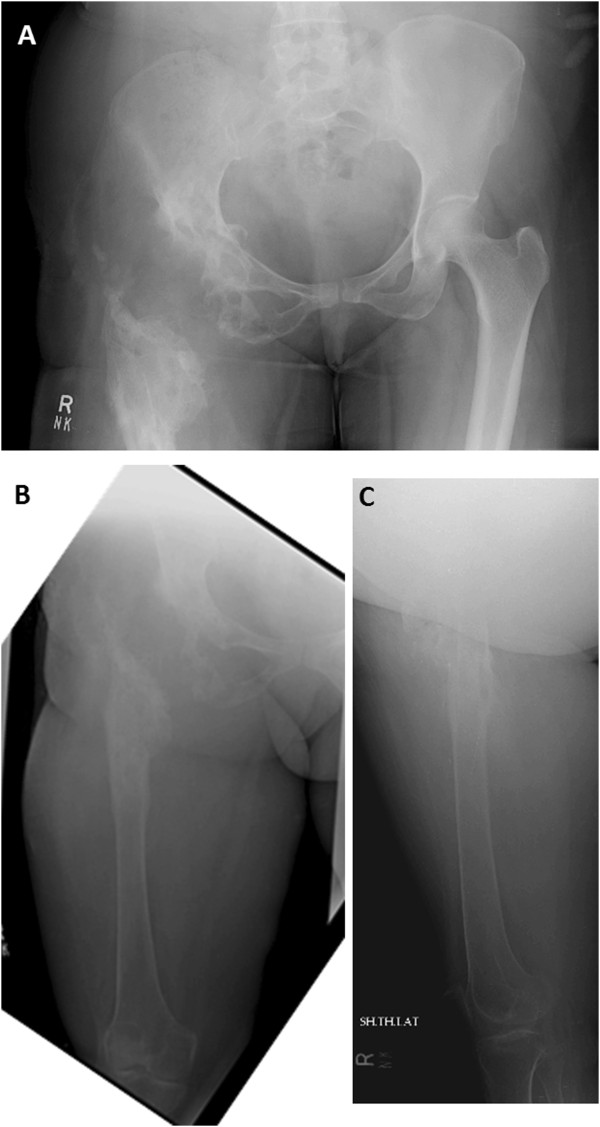
X-ray pelvis (Figure 6A) and femur (Figure 6B and 6C) on presentation at our institute in 2009. There is bone loss in proximal femur with extensive involvement of proximal femur shaft and presence of radiolucencies in acetabulum as well.

**Figure 7:**
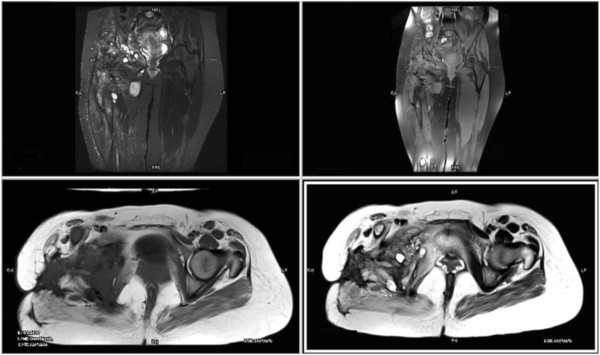
Coronal and axial magnetic resonance images of pelvis show heterogeneous signals on short-tau inversion recovery, predominantly high signals on T2-weighted image (fat saturation) and low signals on T1-weighted image.

**Figure 8:**
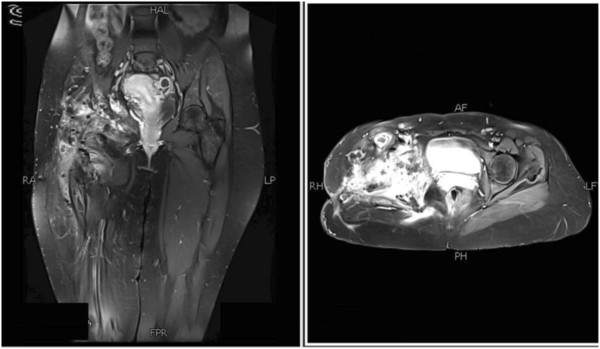
Postgadolinium images showing significant heterogeneous enhancement.

**Figure 9:**
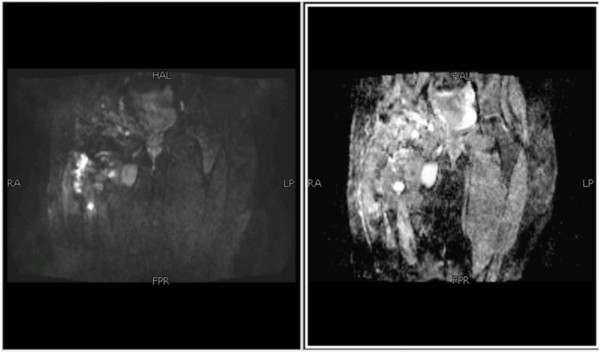
No diffusion restriction on diffusion-weighted imaging and signal drop out on apparent diffusion coefficient mapping.

In 2009, she was offered a hemipelvectomy after which she visited our hospital. On examination she was unable to bear weight on her right leg. Her right leg was shortened by 4cm; the girth of her right thigh was increased by 8cm. There were multiple discharging sinuses over her right thigh and right hip with seropurulent discharge. There were multiple scars over the lateral aspect of her right proximal thigh with limited, painful range of motion of her right hip. Distal neurovascular status was intact. X-rays were performed that showed grossly extensive involvement of her proximal femur and acetabulum (Figures 6A, 6B and 6C). Magnetic resonance imaging (MRI) of her pelvis was done which showed erosions and altered signals in right proximal femur, acetabuli, ischium pubic rami and right ilium (Figures 7, 8 and 9). There was atrophy of her hip muscles, scarring and lymphatics obstruction. Her uterus, ovaries and opposite hip were unremarkable. An ultrasound of her abdomen was unremarkable. Options of hemipelvectomy versus a limb salvage form of modified internal hemipelvectomy and wide margin resection were explained to her. The second option had a risk of recurrence and infection but she opted for the same.

**Figure 10:**
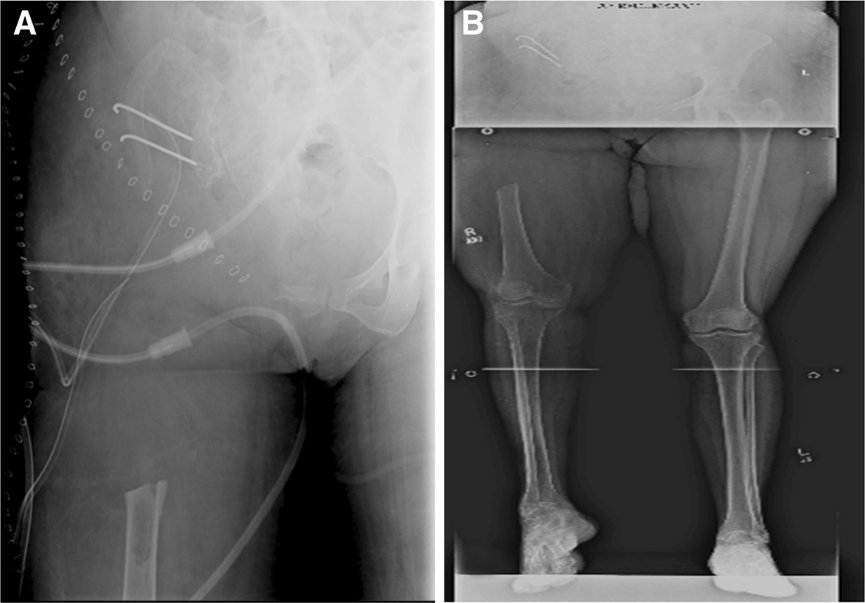
X-ray pelvis (Figure 10 **A**) and lower limb scanogram (Figure 10 **B**) showing internal hemipelvectomy with resection of proximal femur. March 2009.

**Figure 11:**
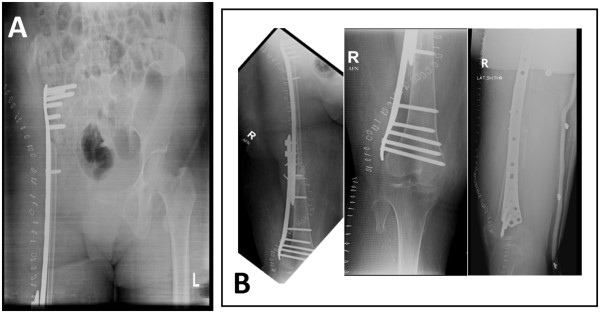
X-ray pelvis (Figure 11 **A**) and femur (Figure 11 **B**) showing reconstruction with free vascularized fibular graft along with LCP (Locking Compression Plate). August 2009.

**Figure 12:**
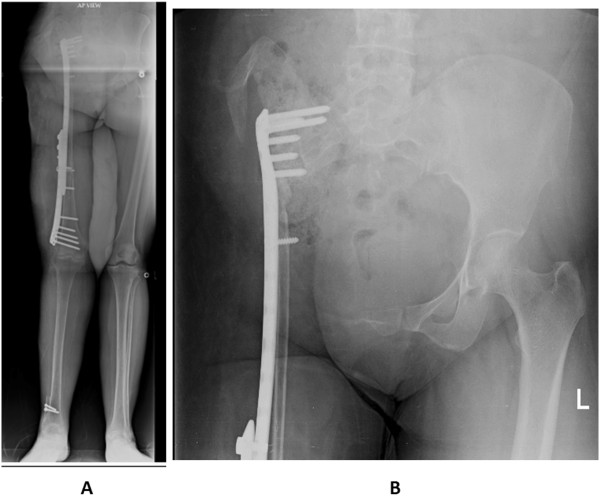
4 month follow-up showing scanogram of lower limbs (Figure 12 **A**) and X-ray pelvis (Figure 12 **B**).

**Figure 13:**
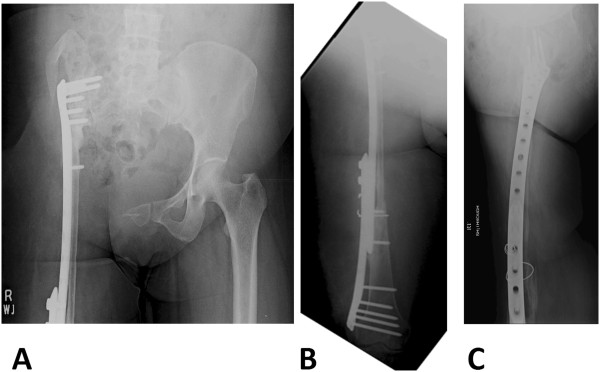
One year follow-up x-rays of pelvis (Figure 13 **A**) and femur (Figure 13 **B** and 13 **C**).

In March 2009, an internal hemipelvectomy with wide margin resection of soft tissue and proximal femur was done (Figures 10A and 10B). Perioperatively frozen sections were sent from resected margins that were reported as disease free (no cyst wall, no scolices). Postoperatively she remained on skeletal traction and received albendazole therapy for more than 3 months. As her wound and infection parameters gradually improved she underwent reconstruction with free vascularized fibular graft along with locking compression plate (LCP) in August 2009 (Figures 11A and 11B). Postoperatively a hip spica was applied. Follow-up imaging was done to assess union (Figures 12A and 12B; Figures 13A, 13B and 13C). Gradually her ambulation progressed and she was able to ambulate without support after a long time.

**Figure 14:**
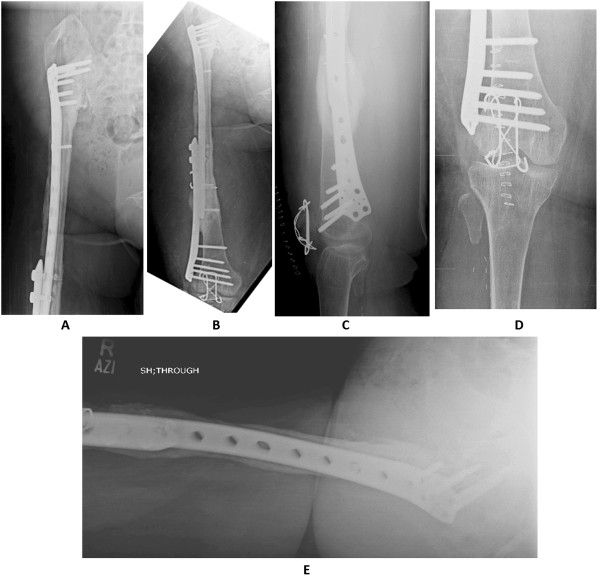
Follow-up X rays of femur (Figure 14 **A** and Figure 14 **B**), knee (Figure 14 **C** and Figure 14 **D**) and lateral view of hip (Figure 14 **E**), at 42 months following surgery, showing good consolidation of union and hypertrophy of the fibula. Also note fixation of patella (Figure 14 **C** and Figure 14 **D**).

She remained ambulant ‘as tolerated’ until February 2013 when she had a fall at home and sustained right patella fracture for which tension band wiring was done (Figures 14A to 14E). Her postoperative recovery remains smooth and now, in August 2014, 60 months (5 years) postoperative from her reconstructive surgery she is ambulant without any support. At present she is infection and disease free.

## Discussion

Hydatid pelvic disease of the bone is considered to be the disease of the young. Diagnosis is primarily based on the findings of X-rays and computed tomography (CT) scans [9–11]. Hydatid bone disease should be considered in any differential diagnosis of osteolytic lesions, especially in endemic regions. The location is mostly hepatic (75%) and pulmonary (15%), and only 10% occur in the rest of the body. Primary skeletal involvement seldom occurs. Bone involvement is seen in only 1 to 2.5% of cases of hydatidosis [12] and surprisingly musculoskeletal lesions of cystic echinococcosis usually occur as isolated findings and without concomitant hepatic or pulmonary involvement [12, 13]. Nevertheless, the involvement of other organs should be ruled out in any patient with bone hydatidosis. The spine is the most common location for about 50% of osseous hydatidosis, followed by pelvis and hip, the femur, the tibia, the ribs and the scapula [14]. Hydatid disease of bone usually remains asymptomatic over a long period, and it is usually detected after a pathological fracture or secondary infection or the onset of compressive symptoms on adjacent soft tissues. The clinical manifestation may take 10 to 20 years to become obvious, since the cyst grows very slowly. The most common radiological manifestation of skeletal hydatid disease is of a lucent expansile lesion with cortical thinning [15]. The CT appearance of a bone lesion is a well-defined, typically multiloculated osteolytic lesion sometimes with coarse trabeculae within it, giving a honeycomb appearance, which is accompanied by expansion of the bone and thinning of its cortex [16]. The MRI signal intensity pattern of the daughter cysts reflects their contents and may vary in cysts that are dead or alive. The production of hydatid fluid stops when they disintegrate at death [17]. MRI is also helpful in delineating the soft tissue extent of the disease. Immunodiagnosis is useful not only in primary diagnosis but also for follow-up of patients after surgical or pharmacological treatment [18]. Detection of circulating *Echinococcus granulosus* antigens in serum is less sensitive than antibody detection, which remains the method of choice [14]. Enzyme-linked immunosorbent assay, indirect hemagglutination antibody assay, latex agglutination test, and immunoblot test are the most commonly used immunological methods. Theoretically, surgery with a broad safety margin is the best treatment for bone hydatidosis [19]; however, most times this recommendation is impossible. For example, in most common sites of hydatid disease of bone, spine and pelvis, radical resection of the lesion is practically impossible [19]. Comparable data have been collected on the outcome of chemotherapy with benzimidazole carbamate (albendazole) that show encouraging results [14]. Albendazole sulfoxide is better absorbed with higher levels of active metabolite in the cysts compared with other benzimidazoles [14]. Treatment with albendazole is effective, but at least one cycle should be given before operation and six or more courses afterwards [14]. In our study, our patient underwent multiple surgeries before presenting to our institute, which reflects the importance of diagnosing the disease early because delay in diagnosis and inadequate management, as happened in this case, leads to further advancement of disease and psychological stress to the patient and his or her family. The natural course of the hydatid disease was explained to our patient including the chances of recurrence of infection. She underwent wide margin excision and stabilization of proximal femur defect with free vascularized fibula along with LCP. She had a follow-up of 60 months and is infection and disease free until now.

## Conclusions

Hydatid bone disease is a rare entity in our part of the world but a careful history and thorough look at the initial images of our patient would have led to the suspicion of pathologic fracture and subsequently early diagnosis of this difficult problem. A second important learning point in this case was the lack of early referral to a center where this difficult problem could have been handled effectively. This could have minimized the physical, mental and financial stress to the patient and her family.

## Consent

Written informed consent was obtained from the patient for publication of this case report and any accompanying images. A copy of the written consent is available for review by the Editor-in-Chief of this journal.

## Authors’ information

Muhammad Shahid Khan: former resident, Department of Surgery, Section of Orthopaedics, Aga Khan University Hospital, Karachi, Pakistan.
